# Tranexamic Acid Use in Obstetric Hemorrhage: Knowledge and Attitude Among Jordanian Obstetricians and Gynecologists

**DOI:** 10.7759/cureus.57360

**Published:** 2024-03-31

**Authors:** Maen Alsumadi, Asma Basha, Amro AlSumadi, Zeina Obeidat, Bashar AbuKhalaf, Abdelrahman Sulieman, Sleman Shuwehdi, Abdullah AlDeffaie, Ahmed AlQaqaa, Aiman Zakaryia

**Affiliations:** 1 Obstetrics and Gynaecology, Epsom and St Helier University Hospitals, London, GBR; 2 Obstetrics and Gynaecology, The University of Jordan, Amman, JOR; 3 Trauma and Orthopaedics, School of Medicine, University of Jordan, Amman, JOR; 4 Obstetrics and Gynaecology, School of Medicine, University of Jordan, Amman, JOR; 5 Obstetrics and Gynaecology, University of Jordan, Amman, JOR

**Keywords:** tranexamic acid, national guidelines, maternal mortality, jordanian obsterician, hemorrhage

## Abstract

Aim: Tranexamic acid (TXA) use in obstetric hemorrhage has been shown to decrease both maternal mortality and morbidity. This study aimed to explore the knowledge and attitudes of Jordanian obstetricians and gynecologists regarding the use of TXA in obstetric bleeding cases, as well as to identify factors that affect decision-making processes and emphasize the significance of TXA in enhancing maternal health outcomes.

Methodology: This study used a cross-sectional design and a structured questionnaire to gather data from a convenience sample of 1000 Jordanian obstetricians.

Results: Most participants used TXA to address obstetric hemorrhage, with medical training being the primary source of knowledge about TXA for (113/166) 68.1% of respondents. Awareness of TXA's potential benefits was high but some misconceptions existed. Approximately (96/166) 57.8% of the participants were aware of the recommended dosage regimen, and (61/166) 36.7% emphasized the importance of timing of administration. Knowledge of potential side effects was notable, with (55/166) 33.1% aware of life-threatening side effects, such as pulmonary embolism and deep vein thrombosis. Concerns regarding barriers to implementation included the absence of strict guidelines (54.8%) and drug availability ( 91/166; 54.8%). However, (64/166) 38.6% expressed confidence in the effective use of TXA for obstetric hemorrhage treatment. The majority of respondents (154/166; 92.8%) considered additional education and training on TXA use to be important in managing obstetric hemorrhage.

Conclusion: Jordanian obstetricians have used TXA in cases of obstetric hemorrhage despite their experience and knowledge based only on limited resources; the need for national guidelines on when and how to use TXA in obstetric practice is of great importance and got vast support from the Jordanian obstetricians.

## Introduction

Obstetric bleeding is a significant concern in maternal healthcare worldwide and is a leading cause of maternal morbidity and mortality [1]. Tranexamic acid (TXA), an antifibrinolytic agent, has gained increasing attention as a potential therapeutic intervention for the adverse outcomes associated with obstetrical bleeding [2]. Despite its established efficacy, the implementation of TXA in obstetric practice varies widely across regions and healthcare settings [3-5]. It is crucial to understand the perspectives of healthcare practitioners regarding the successful implementation of TXA in clinical practice [6]. Jordan has made significant progress in maternal healthcare, achieving a significant reduction in the maternal mortality ratio (MMR) [7]. Obstetrical hemorrhage remains the leading cause of maternal morbidity and mortality worldwide. The World Health Organization recommends the early administration of TXA for all women experiencing postpartum hemorrhage (PPH), emphasizing its life-saving potential [8]. Regrettably, data on the knowledge, attitudes, and practices of Jordanian obstetricians and gynecologists regarding the use of TXA for obstetric bleeding are limited. Furthermore, the factors that influence the decision-making process in this critical context are not well understood [9,10].

This research aimed to explore the knowledge and attitudes of Jordanian obstetricians and gynecologists toward the use of TXA in obstetric bleeding cases, as well as to identify factors that affect decision-making processes and emphasize the significance of TXA in enhancing maternal health outcomes. Furthermore, the study aimed to provide insights that could be used to develop effective interventions. Given the unique healthcare system and cultural factors in Jordan, it is essential to understand the perspectives of healthcare providers regarding the integration of TXA into obstetric care. Addressing obstetric bleeding, which poses a significant threat to maternal mortality, requires tailored interventions in Jordan. Therefore, gaining insight into the perspectives of healthcare providers on TXA is crucial for developing effective strategies to improve maternal health outcomes in this region.

The study had several important objectives, including assessing the current level of knowledge among Jordanian obstetricians and gynecologists regarding the clinical applications, dosages, and potential risks associated with TXA in obstetric bleeding scenarios. Additionally, this study aimed to evaluate the general attitudes of healthcare practitioners toward the incorporation of TXA into obstetric care protocols and explore their perceptions of its efficacy, safety, and feasibility within the local context. The study also aimed to identify the key factors that affect the decisions made by Jordanian obstetricians and gynecologists when administering TXA in cases of obstetric bleeding.

In conclusion, this study aimed to fill a gap in the current literature by examining the comprehensive knowledge and attitudes of Jordanian obstetricians and gynecologists toward the use of TXA for obstetric bleeding. The results of this study are expected to influence healthcare policies, improve clinical guidelines, and contribute to global conversations on enhancing maternal health outcomes through evidence-based interventions.

## Materials and methods

This research was carried out through a cross-sectional study and data were gathered using a structured questionnaire that was specifically designed for Jordanian obstetricians and gynecologists employed in a range of healthcare sectors across the country, including the Ministry of Health, Royal Medical Services, university hospitals, and private practice. The goal of this study was to investigate a particular group of professionals, namely obstetricians in Jordan. To achieve this, 1000 obstetricians were selected from the Jordanian Society of Obstetrics and Gynecology (JSOG) directory using a convenience sampling method. To be included in the study, obstetricians had to fulfill certain criteria, such as being a registered member of the JSOG, possessing a valid email address or WhatsApp number, and agreeing to participate in the study. On the other hand, obstetricians who were retired, not practicing obstetrics, or did not respond to the questionnaire were excluded from the study.

The researchers designed a comprehensive questionnaire, which was pre-tested and consisted of five distinct sections. The first section focused on social demographic characteristics, the second on the awareness and knowledge of respondents regarding the use of TXA in the prevention and treatment of PPH, the third on the type of practices responders had, the fourth on the use of TXA for PPH, and the fifth on the acceptability and barriers to using TXA treatment for PPH.

The questionnaire was distributed to a selected group of obstetricians via WhatsApp messages or emails, depending on their preferred method of communication. Two reminders were sent to non-responders after one and two weeks, respectively, to ensure a high response rate. The response rate was determined by dividing the number of completed questionnaires by the total number of eligible participants.

Informed consent was obtained from all participants, and participation in the study was voluntary. The consent section emphasized the confidentiality and anonymity of the responses, and a cover letter was included with the questionnaire to explain the study's purpose.

Data were sourced from an Excel spreadsheet compiled and maintained internally by the researchers. This spreadsheet contained responses to the questionnaire, and the extracted data were represented by figures to represent the different aspects of the responses and their percentages.

## Results

**Figure 1:**
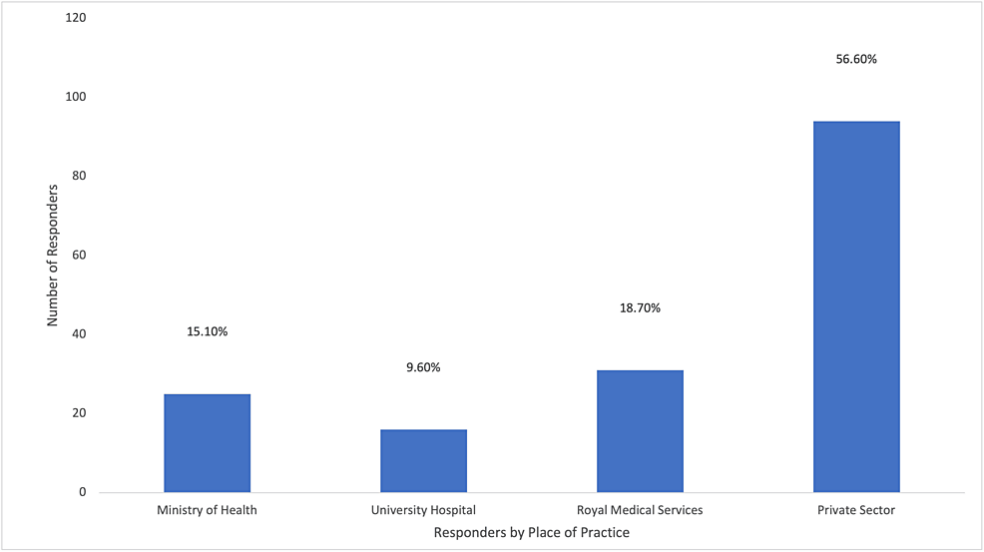
Percentages of Responders According to Their Place of Practice

**Figure 2:**
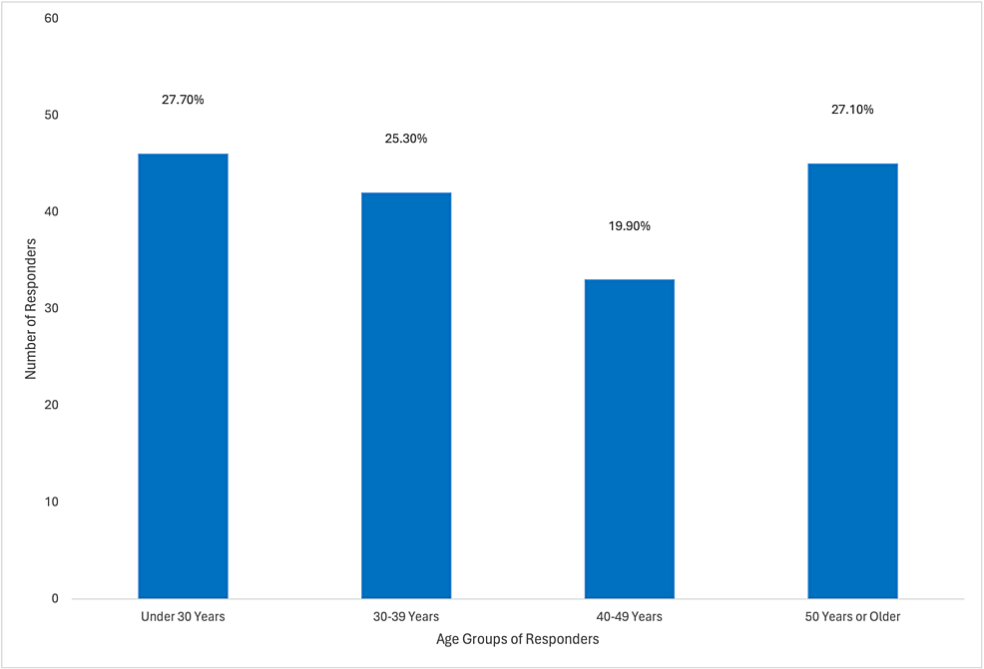
Percentages of Responders According to Their Age Group

**Figure 3:**
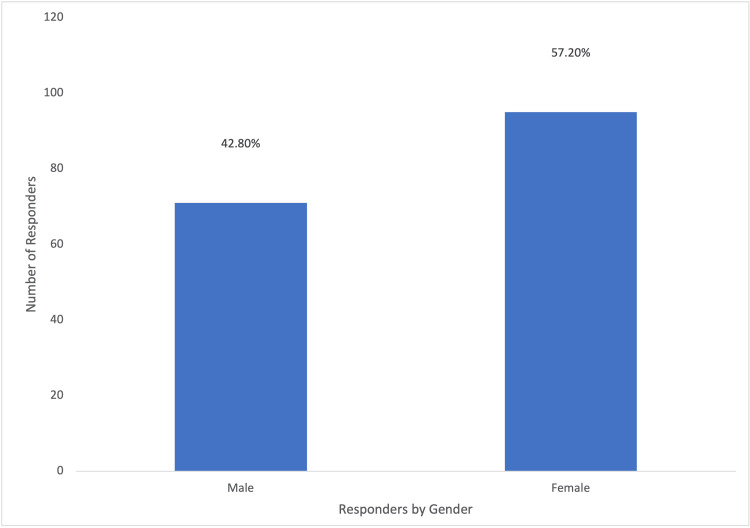
Percentages of Responders According to Their Gender

**Figure 4:**
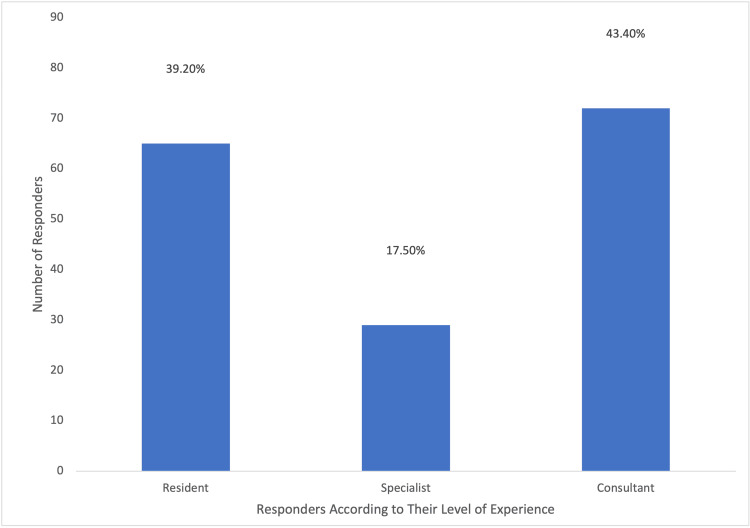
Percentages of Responders According to Their Level of Experience

**Figure 5:**
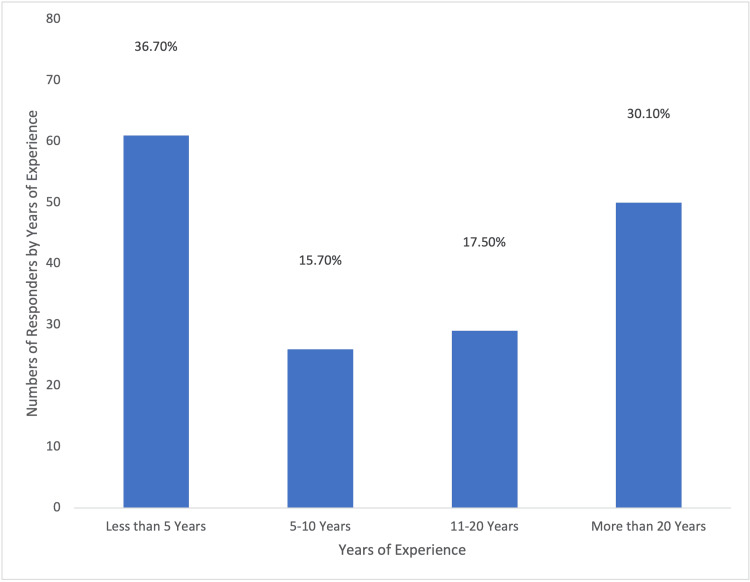
Percentages of Responders According to Numbers of Years of Experience

A total of 166 questionnaires were analyzed in this study, and none were excluded; 90 responses were from private sector individuals, 31 from Royal Medical Services, 25 from the Ministry of Health, and 16 from university hospitals. The distribution of these responses is visually represented in Figure 1. Approximately, one-third of the participants (61/166; 36.7%) had been practicing obstetrics and gynecology for less than five years, and (46/166) 27.7% were under 30 years of age, as illustrated in Figure 2. Additionally, (77/166) 52.4% of the respondents were female, as depicted in Figure 3. In terms of their level of expertise, the majority of the participants (72/166; 43.4%) were consultants, (29/166) 17.5% were specialists, and (65/166) 39.2% were residents, as illustrated in Figure 4. Notably, a larger proportion of participants (79/166; 47.6%) had been practicing obstetrics and gynecology for more than a decade, as illustrated in Figure 5.

**Figure 6:**
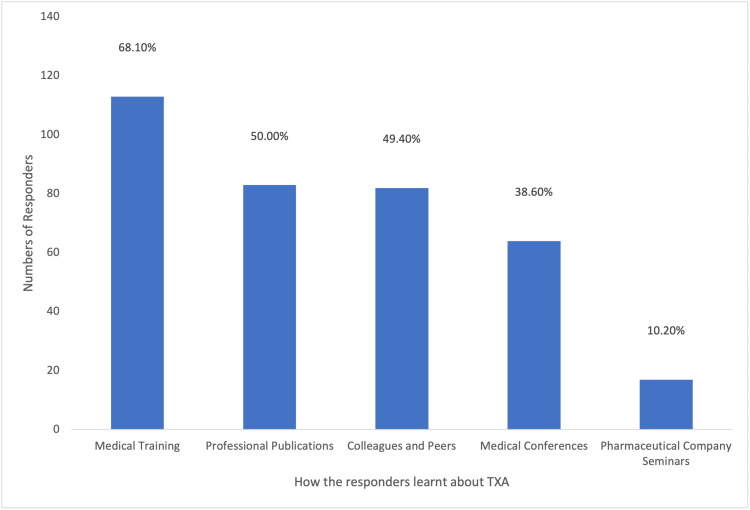
The Ways Participants Learned About TXA TXA, tranexamic acid.

**Figure 7:**
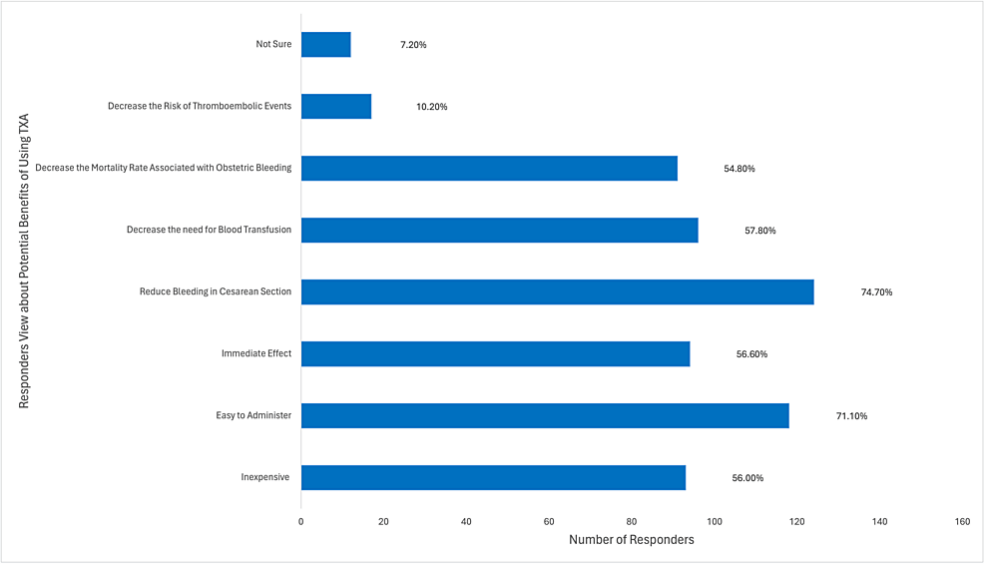
The Participants' Views About the Advantages of TXA Use TXA, tranexamic acid.

**Figure 8:**
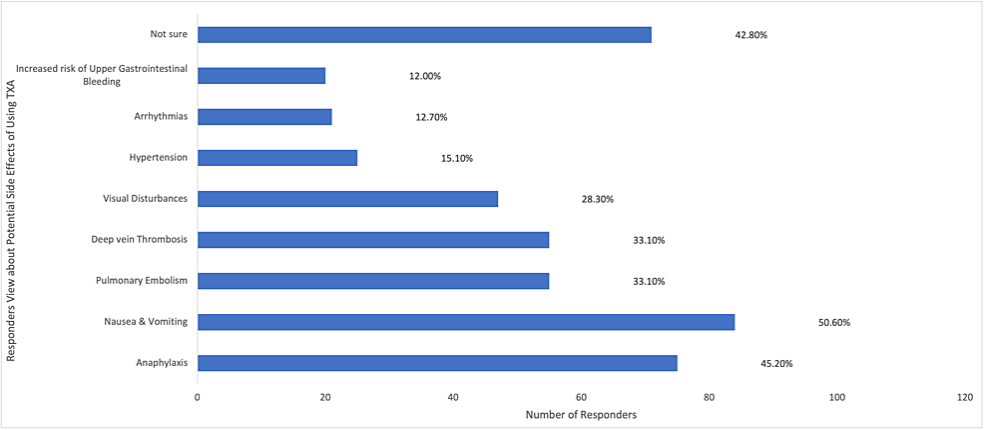
The Participants' Views About the Adverse Effects of TXA Use TXA, tranexamic acid.

**Figure 9:**
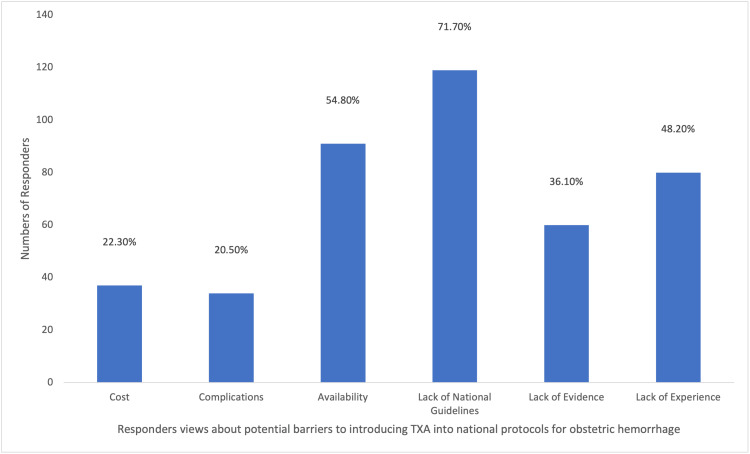
The Participants' Views About the Potential Barriers for TXA Use TXA, tranexamic acid.

**Figure 10:**
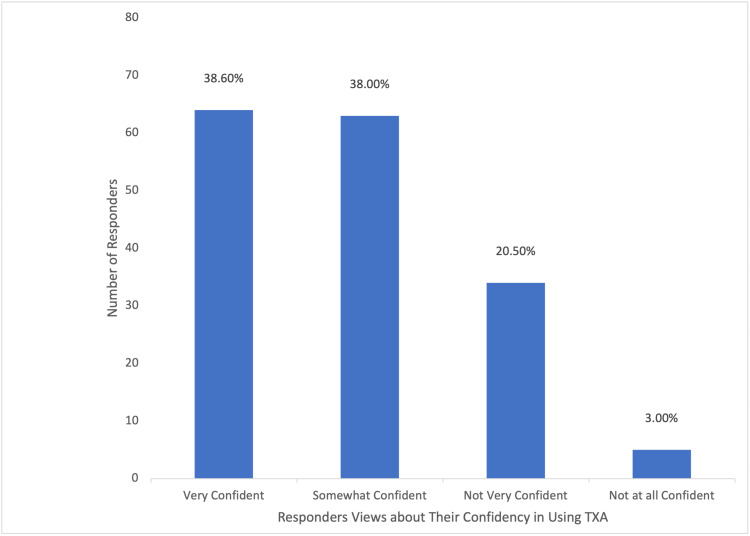
The Participants' Views About Their Confidence Using TXA TXA, tranexamic acid.

**Figure 11:**
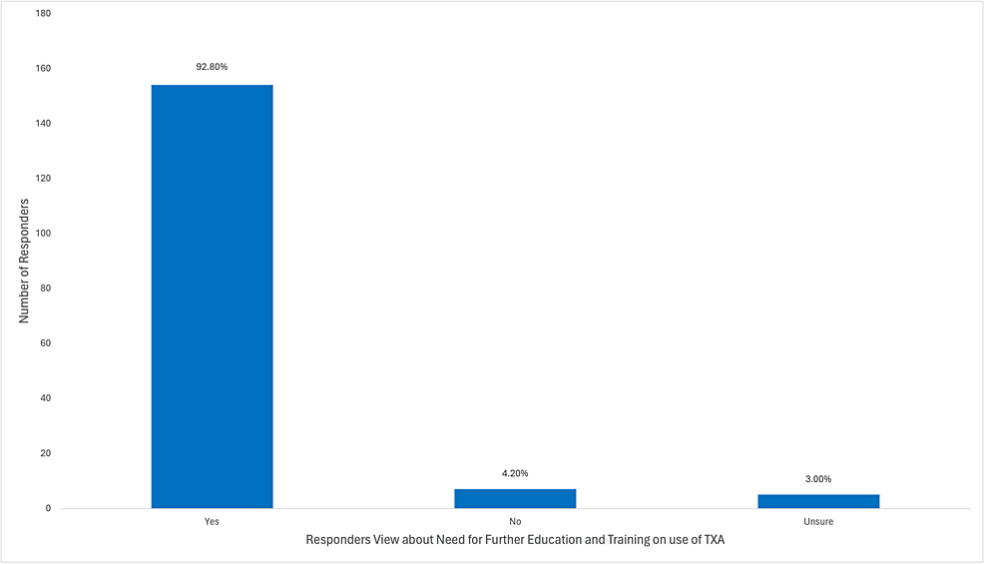
The Participants' Views About the Need for Further Education and Training on TXA Use TXA, tranexamic acid.

**Figure 12:**
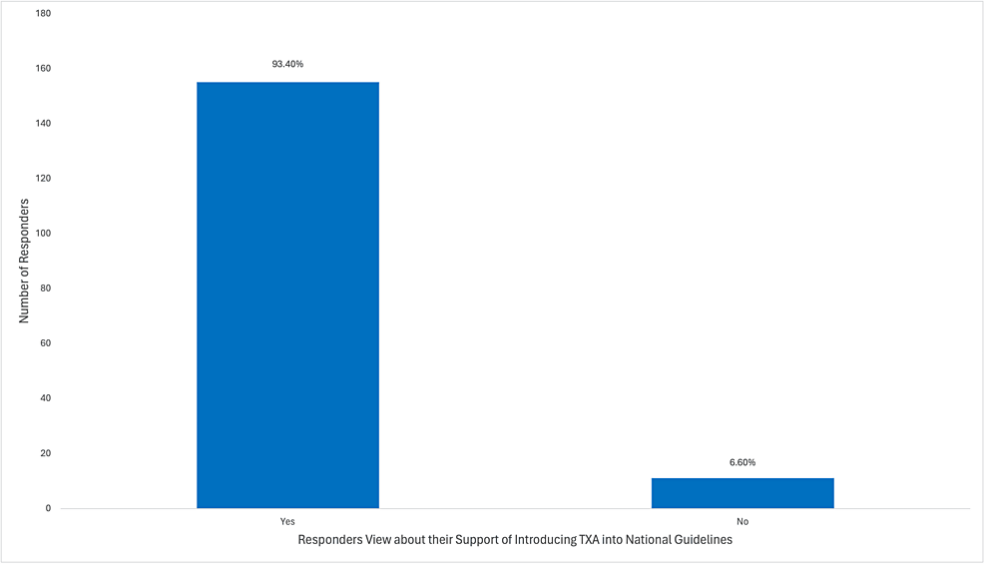
The Participants' Views About the Need to Incorporate Use of TXA in National Guidelines for Management of Obstetric Bleeding TXA, tranexamic acid.

Among the participants, a majority (148/166; 89.2%) utilized TXA to address obstetric hemorrhage, with (77/166) 46.4% frequently employing it in their practice. A substantial portion (113/166; 68.1%) of the participants acquired knowledge about TXA through medical training, as depicted in Figure 6. A considerable proportion (124/166; 74.7%) of the participants were aware that TXA could reduce bleeding during cesarean section, while (91/166) 54.8% were aware of its potential to decrease mortality associated with obstetric hemorrhage. However, (17/166)10.2% of participants believed that TXA decreased the risk of thromboembolic events, as illustrated in Figure 7. Approximately half (96/166; 57.8%) of the participants indicated the recommended dosage regimen of 1 g (100 mg/mL) intravenously over 10 minutes, and (61/166) 36.7% of the participants highlighted the significance of timing in administering TXA, suggesting a range of 1-3 hours after childbirth as the optimal time to administer the drug, which was considered of great importance. Jordanian OBGYNs demonstrated sound knowledge about the potential side effects of TXA usage, as (55/166) 33.1% were aware of the life-threatening side effects of pulmonary embolism and deep vein thrombosis. However, some participants believed that TXA could cause arrhythmias and increase the risk of upper gastrointestinal bleeding (21/166 and 20/166; 12.7% and 12%, respectively), as shown in Figure 8. Regarding the obstacles that Jordanian obstetricians and gynecologists thought would impede the implementation of TXA in national protocols, the primary concern was the absence of strict guidelines for the use of TXA in practice; almost half (91/166; 54.8%) of the respondents identified the availability of the drug as a potential barrier as the drug is not available in all Jordanian hospitals, and some attributed a lack of experience (80/166; 48.2%) as a hindrance, as illustrated in Figure 9. However, (64/166) 38.6% of the participants expressed confidence in their ability to effectively employ TXA in the treatment of obstetric hemorrhage, as shown in Figure 10. The necessity of providing additional education and training to healthcare professionals regarding the appropriate use of TXA for managing obstetric hemorrhage is highlighted by the fact that the majority of respondents (154/166; 92.8%) believe it to be an important issue (as depicted in Figure 11). Furthermore, (155/166) 93.4% support introducing TXA into national protocols for the management of obstetric bleeding (Figure 12).

## Discussion

Obstetric bleeding, which has significant implications on maternal mortality and morbidity worldwide, is a major issue. One severe form of obstetric bleeding, PPH, poses a significant threat to maternal health and accounts for a substantial proportion of maternal deaths and complications. According to the World Health Organization (WHO), PPH is responsible for approximately 27% of all maternal deaths worldwide [11]. This underscores the critical need to recognize obstetric bleeding as a leading cause of maternal and postpartum morbidities.

A study conducted by Say et al. [12] also highlighted the severity of PPH, reporting that approximately 70,000 women die annually due to excessive bleeding during childbirth. Most of these deaths occur in low-income countries, which emphasizes the unequal impact of obstetric bleeding on maternal health, particularly in areas with limited resources.

Furthermore, the consequences of PPH extend beyond mortality, and a substantial number of women experience postpartum complications. A systematic review by Alkema et al. [13] demonstrated that obstetric bleeding, including PPH, significantly contributes to long-term health issues, such as anemia, infections, and impaired reproductive health.

Recognizing the significance of obstetric bleeding in ensuring maternal well-being is essential when developing effective interventions. There are various medical, nonsurgical, and surgical options for treating PPH. TXA is a promising therapeutic option that has demonstrated a 31% reduction in deaths due to bleeding when administered early, as shown by the WOMAN Trial Collaborators [4]. This highlights the potential of TXA to mitigate the adverse effects of obstetric bleeding.

TXA is a vital medication that should be readily available in emergency obstetric care facilities due to its cost-effectiveness, heat stability, and long shelf life. A recent economic evaluation utilizing data from the WOMAN trial demonstrated that administering TXA as standard care for women experiencing PPH in Nigeria and Pakistan is likely to be highly cost-effective [14]. A Cochrane review examining the use of antifibrinolytic agents for treating PPH identified three eligible trials, two of which compared intravenous TXA with placebo or standard care, including the WOMAN trial where 20,060 women were enrolled and the French trial [15,16]. The French trial recruited 152 women with PPH, defined as blood loss greater than 800 mL following vaginal delivery, and randomly allocated them to receive high-dose TXA (loading dose 4 g over 1 hour, then infusion of 1 g/hour over 6 hours) or standard care. A meta-analysis of 20,172 women from these two trials revealed that TXA reduced the risk of death due to bleeding, with early treatment being more effective. Based on this review, the WHO updated its recommendation for the use of TXA in treating PPH, strongly recommending early treatment within 3 h of birth with intravenous TXA using the same dosing regimen as that used in the WOMAN trial: 1 g in 10 mL (100 mg/mL) intravenously at a rate of 1 mL per minute. If bleeding continued after 30 min or restarted within 24 h of the first dose, a second intravenous dose of 1 g was administered. The standard recommendation for women presenting with clinically significant blood loss exceeding 500 mL following a vaginal birth, 1000 mL after a cesarean section, or any other form of bleeding that could compromise hemodynamic stability is to administer TXA. This applies to all causes of bleeding [17].

Recognizing the significance of maternal mortality in Jordan is essential for understanding the need to address obstetric hemorrhage in the country. Obstetric bleeding, particularly PPH, has emerged as a significant contributor to maternal deaths in Jordan, underscoring the necessity for targeted interventions. According to the Jordanian Ministry of Health (MOH), the country's MMR was estimated at 38.5 per 100,000 live births in recent years. In 2019, Jordan's MMR was 32.4 per 100,000 live births, and obstetric hemorrhage accounted for 9.4% of maternal deaths that year, ranking third after COVID-related deaths and thromboembolism. Obstetric bleeding accounted for more than half of the cases in the 2019 report [18].

Addressing the issue of maternal morbidity and mortality resulting from obstetric bleeding in Jordan requires a multifaceted approach that incorporates preventative measures and evidence-based interventions. The Jordanian maternal mortality report emphasizes the significance of "Active Management of the Third Stage of Labor" as a means of preventing PPH. According to a report, public sector hospitals in Jordan have successfully implemented this approach, resulting in a substantial reduction in the number of cases of atonic PPH in recent years. This reduction was likely due to the implementation of numerous interventions. Additionally, the use of TAX has proven to be an effective strategy for managing the fourth stage of labor.

To reduce maternal morbidity and mortality related to obstetric bleeding in Jordan, a comprehensive approach should be adopted that includes enhancing antenatal care, increasing access to skilled birth attendants, and implementing evidence-based interventions such as TXA. By integrating these strategies into existing healthcare frameworks, Jordan can make significant progress toward achieving better maternal health outcomes.

According to Ahmadzia et al. [19], the utilization of TXA in obstetrics and gynecology practice varies across different regions and practices, as demonstrated in their study on the use of TXA during delivery in the United States. The study showed that TXA use increased over time and was more prevalent in the west than in the central and eastern states, with no significant difference in the rate of increase between regions. In contrast, Litman et al. conducted a study in the United States and found that TXA was used in approximately 1% of deliveries, with greater use in the central and southern regions. The results showed a rapid increase in TXA use after 2017, despite a relatively constant number of pregnancies, which may be attributed to the increasing support for its use in preventing PPH [20]. Ahmadzia et al. conducted a study from 2019 to 2021, during which 3.2% of patients received TXA, indicating an increase in usage over time in all regions, with the highest rates in the western region of the United States. The rate of increase varied among the western, central, and eastern regions [21].

This cross-sectional study was conducted among healthcare professionals, specifically doctors, pharmacists, and nurses, in two Nigerian tertiary teaching hospitals. The results revealed that only 23.7% of the participants had adequate knowledge of the proper use of TXA in the management of PPH. Moreover, only 19.8% of the respondents were familiar with the recent WHO recommendations regarding the use of TXA for PPH. It is worth noting that most participants had neither prescribed nor dispensed TXA, and the primary barriers to its use included a lack of awareness of the WHO recommendations, preference for alternative uterotonics, and the cost of the drug [22].

The distribution of respondents across different backgrounds of different practices in Jordan including Public health, private practice, and university hospitals suggests that the use of TXA is not influenced by location or resource availability. The majority of respondents used TXA, indicating that it is not yet a standard practice in Jordan. This could be due to the lack of national guidelines for the use of TXA in obstetric bleeding or general management of obstetric bleeding.

Most respondents learned about TXA through medical training or professional publications, emphasizing the importance of incorporating up-to-date management options in medicine. Although most respondents were able to identify the benefits of using TXA, including reducing bleeding during delivery and decreasing maternal mortality, some (10.2%) identified a decreased risk of thromboembolic risk as a potential benefit. This suggests a limited understanding of TXA's side effects and mechanism of action among some respondents.

The results of the study suggest that a considerable portion of respondents, approximately (66/166) 40%, lack the necessary knowledge regarding the proper dosing and timing of TXA administration. This highlights the need for the development and implementation of clear national guidelines to ensure the safe and effective use of TXA. It is noteworthy that (155/166) 93.4% of the respondents endorsed the importance of these guidelines, underscoring their value in improving the quality of care and achieving the desired outcomes.

To ensure that obstetricians in Jordan possess the necessary knowledge and skills regarding TXA, it is imperative to introduce educational initiatives, such as workshops, training programs, and online modules. These initiatives should aim to address the lack of awareness, dispel misconceptions, and emphasize the appropriate use of TXA in various obstetric bleeding scenarios. It is essential that these efforts be evidence-based, culturally sensitive, and tailored to the specific needs and contexts of Jordanian healthcare practices.

Moreover, the development and implementation of clear, concise, and updated national guidelines for TXA use in obstetrics are crucial for enhancing clinical practice. These guidelines should be based on international best practices, adapted to the local context, and readily accessible to all Jordanian obstetricians. Regular updates and dissemination efforts are necessary to ensure adherence and promote consistent life-saving interventions. The implementation of such guidelines has demonstrated significant benefits in reducing the need for interventions to treat postpartum bleeding, as demonstrated by a study conducted by Watikens et al., although they did not show benefits regarding other parameters, such as postpartum anemia [23].

Limitations to our study might include response bias; in our study, we collected a response rate of 16.6% from the 1,000 questionnaires distributed. This may suggest a potential bias in the sample. Respondents who had prior experience with TXA were more likely to participate, while those who had no experience were less likely to do so. This is evident from the fact that 89.2% of the respondents reported having used TXA before, and most of them had been practicing obstetrics for more than five years, indicating their familiarity with the medication.

Another limitation is the lack of decision-makers' perspectives regarding the advantages and barriers to introducing the use of TXA in national guidelines. 

## Conclusions

The use of TXA in treating obstetric hemorrhage in Jordan has been embraced by obstetricians, despite their constraints in terms of resources and expertise. It is widely recognized that the development of national guidelines for the appropriate use and timing of TXA in obstetric care is of paramount importance. This has been met with widespread support from Jordanian obstetricians, who acknowledge the need for such guidelines.
